# Appearance Teasing and Mental Health: Gender Differences and Mediation Effects of Appearance-Based Rejection Sensitivity and Dysmorphic Concerns

**DOI:** 10.3389/fpsyg.2019.00579

**Published:** 2019-03-20

**Authors:** Jennifer Schmidt, Alexandra Martin

**Affiliations:** ^1^Clinical Psychology and Psychotherapy, School of Human and Social Sciences, University of Wuppertal, Wuppertal, Germany; ^2^Department of Psychology, HSD Hochschule Döpfer University of Applied Sciences, Cologne, Germany

**Keywords:** appearance teasing, mental health, body image, gender differences, body dysmorphic disorder, appearance-based rejection sensitivity

## Abstract

Appearance teasing is a common phenomenon in social interactions, especially in adolescence. Several studies have shown its negative impact on mental health as well as on body image. While these findings prove stable in various contexts, less evidence is available for possible gender differences in these relationships. In particular, the role of two important body image variables – appearance-based rejection sensitivity (ARS) and dysmorphic concerns – and their contribution to mental health impairments has not been assessed in gender-specific process models. In a cross-sectional survey-study (*N* = 501; 407 f, 94 m), we retrospectively assessed early appearance teasing experiences, as well as current ARS, dysmorphic concerns, depression, anxiety, and self-esteem. We analyzed gender differences in these variables and their interrelations. We then examined the mediating role of ARS and dysmorphic concerns in explaining mental health variables in adulthood due to early appearance teasing in gender-specific serial-mediation models. The results show high ratios of early teasing experiences, but no significant gender difference regarding the frequency of early appearance teasing. While teasing experiences were significantly related to body image variables in adulthood in both genders (*r*s > 0.32; *p*s < 0.010), we observed significant relations with mental health outcomes in women (*r*s > 0.30; *p*s < 0.001) but not in men (*r*s < 0.20; *p*s > 0.250). Serial mediation models show that ARS and dysmorphic concerns mediate the effects of appearance teasing on mental health in all outcomes in women (Δ*R^2^* > 0.17), but not in men (Δ*R^2^* < 0.03). Findings remained stable when controlling for Body-Mass-Index, age, and relationship-status. The findings show similar frequencies of appearance teasing and associated negative effects on body image in men and women. Specifically, in women, the effects of teasing on mental health were stronger and mediated by ARS and dysmorphic concerns. Overall, the results point to the relevance of ARS for etiological models of body image disorders and female mental health. However, men did not show the same relationships of teasing and mental health. Differential resilience factors regarding the negative effects of early appearance teasing could be an important target for future research.

## Introduction

Appearance teasing (i.e., negative social feedback on one’s physical characteristics; Cash, 1995) is a common phenomenon, to which a great number of individuals is exposed during childhood and adolescence. It often occurs in the form of verbal harassments and provocations, ranging from name-calling (e.g., “fatso,” “pizza face,” or “wimp”) to hostile remarks. In this context, teasing needs to be distinguished from victimization and bullying behaviors with a more physical and violent nature, such as sexual harassment, severe threats, or physical violence (e.g., Kochenderfer and Ladd, 1996; Keltner et al., 2001).

Studies on the prevalence, types, and effects of appearance-related teasing experiences show that approximately three-quarters of college students reported such teasing experiences (Cash, 1995; Hayden-Wade et al., 2005; Almenara and Ježek, 2015). Regarding the target of teasing, experiences often relate to the individual’s body weight (e.g., Cash, 1995; Hayden-Wade et al., 2005). However, appearance teasing can also relate to facial features (e.g., the nose), body height, chest, hair, or extremities (Cash, 1995). Sources of teasing experiences are peers (up to 60%), but also siblings (up to 36%) and parents (up to 19%) (Cash, 1995; Keery et al., 2005).

There is inconsistent evidence with regard to gender-specific occurrence of appearance teasing. For example, some studies report higher teasing frequencies in girls compared to boys (Farrow and Fox, 2011; Almenara and Ježek, 2015). Other studies report higher rates of verbal bullying and appearance pressure reported by boys compared to girls (Jones and Crawford, 2006; Stubbs-Richardson et al., 2018) or no gender differences at all (Schwartz et al., 1999; Phares et al., 2004). These contradictory results might emerge from a great heterogeneity in studied samples with respect to age, cultural background, and different conceptualizations of teasing (e.g., Liang et al., 2011).

While the numbers suggest that appearance teasing is a common and widespread phenomenon, it would be a mistake to discount its meaning and severity. Many studies with focus on the examination of appearance teasing showed negative effects on several mental health outcomes. These encompass impairments in self-esteem, increased levels of depression (Jackson et al., 2000; Faith et al., 2008; Greenleaf et al., 2014; Feragen and Stock, 2016), and even suicidal ideation and attempts (Eisenberg et al., 2003; Ford et al., 2017). In adolescents, teasing experiences are associated with social avoidance, fear of negative evaluation, and loneliness (Storch et al., 2003). McCabe et al. (2010) reported associations of retrospective accounts of teasing during childhood and anxiety disorders in adults, especially social anxiety. In addition, disordered eating, psychosomatic problems, and physical health show relations with appearance teasing experiences (Keery et al., 2005; Gini and Pozzoli, 2009; Thompson et al., 2018). Overall, the negative impact of general types of victimization on psychosocial adjustments (operationalized as depression, anxiety, self-esteem) is well established (Hawker and Boulton, 2000). While appearance teasing thus poses a high risk of negative outcomes in individuals reporting teasing, evidence on possible gender differences is - again - less consistent. Some researchers reported stronger and broader negative outcomes of teasing in girls (Jones et al., 2005; Gruber and Fineran, 2008; Carbone-Lopez et al., 2010; Feragen and Stock, 2016), while others found gender-invariant effects (Eisenberg et al., 2003).

With regard to the more proximal psychological consequences, adolescent appearance teasing experiences have significant effects on body image concerns (Menzel et al., 2010). Two important constructs that have been highlighted in this regard are the cognitive processes of *dysmorphic concerns* and the personality disposition of *appearance-based rejection sensitivity* (ARS).

Dysmorphic concerns are a core symptom of body dysmorphic disorder (BDD) and describe excessive and time consuming cognitive preoccupation with one’s appearance (Phillips, 2005; American Psychiatric Association, 2013). Empirical findings from cross-sectional and longitudinal studies show that early appearance teasing experiences are associated with more dysmorphic concerns (Lavell et al., 2014; Webb et al., 2015; Weingarden and Renshaw, 2016; Zimmer-Gembeck et al., 2018). In addition, patients diagnosed with BDD retrospectively report higher frequencies of appearance teasing than healthy controls (Buhlmann et al., 2007; Buhlmann et al., 2011; Weingarden and Renshaw, 2016; Weingarden et al., 2017). Correspondingly, etiological models of BDD consider appearance teasing as a risk factor for the development of this mental disorder (Veale, 2004; Neziroglu et al., 2008). BDD symptoms in turn relate to poor mental health, with increased degrees of depression, anxiety, and low self-esteem (e.g., Biby, 1998; Bohne et al., 2002).

As another proximal factor, appearance teasing can influence ARS – the anticipation or fear of social rejection because of one’s appearance. ARS describes the personality disposition to readily expect and be concerned about interpersonal rejection because of one’s physical attractiveness (Park, 2007). Appearance teasing experiences are related to higher levels of ARS (Lavell et al., 2014; Webb et al., 2015, 2017). In itself, ARS is related to depression, anxiety, and low self-esteem (Park et al., 2010; Bowker et al., 2013; Schmidt and Martin, 2017). Further, as a personality disposition, ARS predicts excessive body image concerns (Park et al., 2009; Calogero et al., 2010), is elevated in individuals with BDD (Kelly et al., 2014; Schmidt and Martin, 2017), and explains unique variance in BDD symptom severity in comparison to general rejection sensitivity (Kelly et al., 2014). Recent studies provided increasing evidence for the particular relevance of ARS as a mediator between the effects of appearance teasing and dysmorphic concerns (Lavell et al., 2014; Webb et al., 2015, 2017; Densham et al., 2017).

Given the significant relationship of appearance teasing with negative mental health outcomes as well as their strong effects on body image, a serial indirect mediation effect (i.e., a mediation via two or more mediators that are causally and closely associated due to theoretical conceptions and/or empirical findings) of current ARS and dysmorphic concerns might be present. In the relationship of these two body image variables, the personality disposition of ARS should precede the cognitive processes of dysmorphic concerns, as previous studies have established (Lavell et al., 2014; Webb et al., 2015; Densham et al., 2017). Thus, we determined the order of the assumed mediation as: retrospective appearance teasing → current ARS → current dysmorphic concerns → current mental health.

With regard to gender differences in this pathway model, evidence is fragmented and inconclusive. Menzel et al. (2010) did not find gender differences regarding the associations of appearance teasing with body image in their meta-analysis. In addition, Webb et al. (2015) did not identify moderating influences of gender on the effects of teasing on BDD symptoms. However, some evidence suggests gender differences in specific parts of the assumed mediation model, especially regarding the influence of body image variables on mental health. Vogt Yuan (2010) has shown that body perceptions were associated with changes in psychological well-being in a group of female, but not of male adolescents. Other researchers found that self-esteem of young males was not significantly affected by body image and that boys seem to be less sensitive to teasing in general (Bolognini et al., 1996; Gleason et al., 2000; Furnham et al., 2002; Baudson et al., 2016). With regard to depression as another mental health marker, weaker associations with body image in boys compared to girls have been reported (Marcotte et al., 2002; Hyde et al., 2008). These gender differences might be caused by differential importance of appearance for the overall self-evaluation of male and female individuals, for example, due to media-conveyed beauty ideals (e.g., Lopez-Guimera et al., 2010) and the gender-specific importance of appearance for self-esteem (e.g., Gentile et al., 2009). However, many studies examining appearance teasing effects on body image focused on female samples (Menzel et al., 2010) and even in mixed samples, researchers mostly did not specifically address gender differences (e.g., McCabe et al., 2010).

The aim of the present study was therefore twofold: Firstly, we aimed at examining general gender differences in retrospective appearance teasing experiences in childhood and adolescence, current body image, and mental health because of the ambiguous existing results. In line with previous studies on mental health and teasing (e.g., Hawker and Boulton, 2000; Eisenberg et al., 2003), we operationalized mental health via assessments of depression, anxiety, and self-esteem. In addition, we intended to examine the bivariate relationships between these variables and possible gender differences in the strength and significance of the associations.

Secondly, we aimed at analyzing the postulated mediation effect, that is, whether ARS and dysmorphic concerns mediate the relationship of retrospective appearance teasing in childhood and adolescence and current mental health problems. Based on the results of earlier research on mediation effects in single parts of the hypothesized relationships (Lavell et al., 2014; Webb et al., 2015; Densham et al., 2017), we assumed a serial mediation model (appearance teasing → ARS → dysmorphic concerns → mental health). We performed separate gender-specific model analyses to identify whether indirect effects differ between male and female subsamples due to possible gender differences in the relation of body image variables and mental health (e.g., Gleason et al., 2000; Furnham et al., 2002; Hyde et al., 2008; Vogt Yuan, 2010). Altogether, the small number of existing studies does not allow to state definite hypotheses regarding size and significance of gender-specific indirect effects. Therefore, we conduct the gender-separated mediation analyses in an exploratory approach.

To assure statistical robustness of the findings, we reanalyze the models while controlling for possible confounding variables that have shown strong associations with teasing and mental health in earlier research: (1) Body-Mass-Index (BMI; e.g., Hayden-Wade et al., 2005; McLaren et al., 2008), (2) age (e.g., Kessler et al., 2010), and (3) relationship status (e.g., Willitts et al., 2004).

## Materials and Methods

### Study Design

The cross-sectional study was conducted online via survey in a German-speaking community sample, using the platform *SoSci Survey* (Leiner, 2018). Data collection took place from April 4th to May 18th 2018. The study was conducted in line with the Helsinki Declaration and was approved by the research ethics committee of the University of Wuppertal. The survey was announced as a study on appearance, body image, dermatological conditions, and mental health. Aside from the research questions that are addressed in this report, the study also served for the psychometric evaluation of a questionnaire assessing skin picking, which will be the subject of another manuscript planned. The present report is the first presenting results from the project.

### Participants

The target sample was a German-speaking community sample with a broad range of participants regarding age, professions, and health status. Therefore, the only inclusion criteria for study participation were legal age (≥18 years) and sufficient knowledge of the German language. All participants provided informed consent on the study participation.

We conducted the recruitment via the local university-newsletter, other student newsletters, flyers, and social media-websites (*Facebook*, *Xing*, *What’s App*-groups, *Kleiderkreisel.de*), and via the website of a German magazine on psychological topics (*Psychologie heute*). Psychology students at the University of Wuppertal could receive course credit for participation. All other respondents could participate in a raffle of ten 10 EUR gift-cards.

Power calculations with G^∗^Power 3.0 (Faul et al., 2007) showed that the detection of between-group gender differences with small to medium effect sizes (*d* = 0.35; see Hawker and Boulton, 2000) would require a minimum sample size of *N* = 204, given a significance level of α = 0.05 and statistical power of 1-β = 0.80. For regression-based mediation analyses, *a priori* power calculation resulted in a sample size of *n* = 77 (per group) necessary to detect medium effects (*f*^2^ = 0.15) at the same significance and power levels in linear regression models with three predictors. Allowing for additional dropout and exclusion-margins, we aimed at recruiting an overall sample of *N* > 300.

### Assessment Instruments

#### Sociodemographic Data

To assess sociodemographic characteristics of the sample, the questionnaire included items on the participant’s gender (*male*/*female*/*other*), current age (in years), current body weight (in kg) and height (in cm) for the calculation of the BMI (in kg/m^2^). Further questions assessed the current relationship status in a binary format (*yes* / *no*), the level of school education, and the current employment status.

#### Appearance Teasing Experiences

To assess appearance teasing, an initial question asked whether the participants had ever experienced any severe teasing or bullying/mobbing throughout their lives (*yes*/*no*).

Appearance-related teasing was then assessed with a modified version of the weight-teasing subscale of the *Perceptions of Teasing Scale (POTS*, Thompson et al., 1995; German version: Losekam et al., 2017). The POTS is a commonly used measure to retrospectively assess teasing experiences during childhood and adolescence in adult samples (for an overview, see Menzel et al., 2010). The adapted subscale assesses the frequency and effects of *appearance*-related teasing experiences in childhood and adolescence. It consists of six items that ask about the teasing experiences while growing up (age 5–16). For each item, respondents can use 5-point Likert scales to indicate (a) the frequency of described situations (1 = *Never*, 3 = *Sometimes*, 5 = *Very often*) and (b) the effects (“How upset were you?” 1 = *Not upset*, 3 = *Somewhat upset*, 5 = *Very upset*). Separate mean scores on teasing frequency (WT-F) and teasing effects (WT-E) indicate more frequent teasing-experiences and stronger effects of these experiences (Thompson et al., 1995). The POTS has good psychometric properties, with a good internal consistency (Cronbach’s α > 0.87), high test-retest-reliabilities (WT-F: *r_tt_* = 0.90; WT-E: *r_tt_* = 0*_._*85), and evidence for its concurrent validity in adult and preadolescent samples (Thompson et al., 1995; Jensen and Steele, 2010).

For the purpose of the present study, whenever the original items referred to weight as the cause of the teasing, we replaced the respective words with more general terms that address the overall appearance (e.g., “People made fun of you because you were heavy” was changed to “People made fun of you because of your appearance”). We only used the teasing frequency (POTS-TF) subscale for analyses. The internal consistency of the POTS-TF was good in the present sample (α = 0.89).

#### Appearance-Related Mediators

##### Dysmorphic concerns

We assessed dysmorphic concerns with the Dysmorphic Concerns Questionnaire (DCQ; German version: Stangier et al., 2003). The DCQ is a frequently used screening instrument to assess cognitive appearance concerns and symptoms of BDD via self-report. The scale consists of seven items to be answered on 4-point Likert scales (0 = *not at all*; 3 = *much more than other people*). Here, higher values of the sum score indicate more dysmorphic concerns. The one-factorial instrument has a good reliability and validity (Schieber et al., 2018), and showed a good internal consistency in the present sample (α = 0.85).

##### Appearance-based rejection sensitivity

The personality disposition of ARS was assessed with the Appearance-Based Rejection Sensitivity Scale (ARS-D; Park, 2007; German version: Schmidt and Martin, 2017). The short-form of the ARS-D consists of twelve items that describe brief scenarios related to one’s appearance in various types of social contexts, for example, “You are leaving your house to go on a first date when you notice a blemish on your face.” For each scenario, respondents can use 6-point rating scales to indicate (a) their worries about rejections due to the appearance in the scenario (affective component; 1 = *very unconcerned*; 6 = *very concerned*) and (b) the estimated likelihood of such rejection experiences (cognitive component; 1 = *very unlikely*; 6 = *very likely*). The scores of the affective and the cognitive component of each scenario are multiplied and averaged, leading to a mean score with a range of 1 to 36. Higher values indicate stronger ARS. Psychometric properties of the ARS-D are good with high internal consistency (α = 0.90; Schmidt and Martin, 2017; α = 0.92 in the present sample) and convergent and discriminant validity (Park, 2007; Schmidt and Martin, 2017).

#### Mental Health Outcomes

Three different constructs served as multifactorial indicators of the current state of respondents’ mental health: depression, anxiety, and self-esteem. The respective questionnaires were based on self-report.

##### Depression

We applied the German version of the Patient Health Questionnaire-9 Depression module (PHQ-9; Gräfe et al., 2004) to assess current depressive symptoms. The PHQ-9 contains nine items to rate current symptoms of major depression in line with the respective diagnostic criteria with regard to the previous 2 weeks. The frequency of symptoms is answered on a 4-point rating scale (0 = *not at all*; 3 = *nearly every day*), and the resulting sum score indicates the severity of depressive symptoms. The PHQ-9 is a widely used and valid screener to assess depressive symptoms (Martin et al., 2006) and shows a high internal consistency (α = 0.88, Gräfe et al., 2004; α = 0.89 in the present sample).

##### Anxiety

For the assessment of current symptoms of anxiety, we applied the anxiety module of the Patient Health Questionnaire; the Generalized Anxiety Disorder Scale (GAD-7; Löwe et al., 2008). The GAD-7 assesses typical symptoms of anxiety (e.g., worries, tension, difficulties to relax etc.) on seven items regarding the last 2 weeks. Answers are provided on 4-point rating scales (0 = *not at all*; 3 = *nearly every day*). The overall sum score indicates the severity of anxiety symptoms. The brief economic instrument has shown factorial and construct validity, as well as a very good to excellent reliability (α = 0.88, Löwe et al., 2008; α = 0.90 in the present sample).

##### Self-esteem

Self-esteem was assessed with the German version of the Rosenberg Self Esteem Scale (RSES; von Collani and Herzberg, 2003). The ten-item questionnaire assesses global self-esteem as a stable trait, using 4-point rating scales (0 = *strongly disagree*; 3 = *strongly agree*). After recoding inverted items, the score is summed up with higher values indicating higher self-esteem. The psychometric properties of the instrument are good and correlations with other mental health outcomes are significant (von Collani and Herzberg, 2003; Sinclair et al., 2010). Internal consistency is reported as very good (α = 0.85, von Collani and Herzberg, 2003), with even higher reliability in the present sample (α = 0.91).

### Procedure

Volunteers were able to participate in the study via clicking on a respective hyperlink that was distributed in the recruitment process. They first had to read the study information and provide informed consent. The questionnaire then started with an assessment of the sociodemographic characteristics. This assessment was followed by questions about dermatological problems and skin picking behaviors, to answer another research question. Then, the ARS-D, POTS, RSES, DCQ, GAD-7, and PHQ-9 were administered in the stated order. Intermittently, we included motivational messages to retain the participants’ attention and involvement. A progress bar provided an overview on the percentage of completed questions.

Overall, it was possible to skip items if the respondents were not willing to answer the questions. In this case, a pop up asked whether the items had been left out on purpose. This procedure was chosen to avoid dropout because of reluctance to answer certain sensitive questions. At all time, a contact e-mail address was displayed in case of any further questions or the need to contact the researchers. Overall, it took approx. 20–30 min to fill in the complete questionnaire.

### Data Processing

A total of *N* = 838 respondents first opened the survey website, whereof *N* = 532 completed the survey (i.e., response at the final webpage; dropout rate: 36.5%). We deleted the data of participants who indicated an age <18 or no age at all (*n* = 2), in line with the inclusion criteria of the study. We screened the provided response time measures and normed data quality measures provided by the online software (DEG_TIME; Leiner, 2018) for suspicious working speed (TIME_SUM criterion; one third of the average time in test runs; <9 min) and low data quality (DEG_TIME criterion <100) when filling in the questionnaire. We excluded *n* = 25 datasets due to insufficient data quality. Because of the research question of this study and the small number of individuals who indicated another gender than male or female (*n* = 3), we excluded these cases from the analyses. Another participant was excluded because she did not indicate a weight or height for the calculation of the BMI as a control variable.

Altogether, the amount of missing values in the overall survey was <0.15%. There were no datasets with more than 50% of missing values in any of the applied assessment instruments. For other single missing values, we conducted the *Missing Completely at Random-Test* (MCAR; Little, 1988). The test indicated that there were no systematic patterns in the missing values. We then used a multiple imputation technique (*m* = 20) to replace missing values. The resulting estimates for all 20 imputed datasets were manually compared to the means in the original dataset for all 123 variables with missing values. We aimed at selecting the imputed dataset with the least deviation from the means in the original dataset. Imputation 11 showed deviations from the original dataset in only 17 variables. In 14 of these variables, the deviation from the original mean was 0.01 points and in the remaining three variables, the deviation was 0.02 points, indicating very small deviations. The data were screened regarding extreme outliers in the relevant variables, using box plot analyses. However, no participant had to be excluded for this reason. The final analysis sample consisted of *N* = 501 datasets, with *n* = 407 female and *n* = 94 male respondents.

### Statistical Analyses

Sample characteristics regarding the sociodemographic data and former teasing experiences were calculated as frequencies or means of the female and male subgroups and compared between genders by means of *t*-test and χ^2^-tests. We also performed gender comparisons for the means of all relevant variables in analyses (POTS-TF, DCQ, ARS-D, PHQ-9, GAD-7, RSES) with *t*-tests. However, because Kolmogorov-Smirnov tests and Levene’s tests showed that the assumptions of normality and homoscedasticity were violated for the majority of variables, we backed up the results of those variables with non-parametric Mann-Whitney-*U*-Tests.

We then calculated two-sided correlations to analyze the general relationship between teasing (POTS-TF), mediator variables (DCQ, ARS-D), and mental health outcomes (PHQ-9, GAD-7, RSES) separately for the male and female subgroups, using Spearman correlations with Bonferroni-corrections to adjust for multiple comparisons. Fisher’s *Z*-tests served to analyze potential gender differences in the size of the resulting correlation coefficients of the POTS and the other outcomes.

**FIGURE 1:**
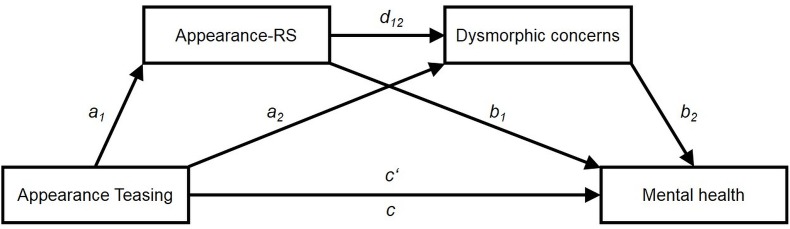
Conceptual mediation model. Appearance-RS, appearance-based rejection sensitivity.

Mediation analyses were conducted with PROCESS for SPSS (Hayes, 2013), using a serial mediation model (model 6) with retrospective teasing frequency (POTS-TF) as predictor, two serial mediators (ARS-D → DCQ), and mental health variables as outcomes (PHQ-9, GAD-7, RSES). The conceptual model (see Figure 1) is based on three linear regression analyses. The first regression analysis tests the effects of appearance teasing frequency (POTS-TF) on ARS (ARS-D values; path a_1_ in Figure 1). The second regression model tests the combined predictive effects of appearance teasing and ARS on dysmorphic concerns (DCQ values; paths a_2_ and d_12_ in Figure 1). The third regression predicts the mental health outcome (PHQ-9, GAD-7, RSES) by the independent variable (POTS-TF) and the two mediators (paths b_1_, b_2_, and c’ in Figure 1). Here, path c’ in Figure 1 depicts the direct effect of appearance teasing on the mental health outcomes controlled for the effects of the two mediators. In contrast, path c in Figure 1 indicates the total effect of appearance teasing on mental health outcomes without considering the mediators.

For each mental health outcome, we calculated separate models based on the male and female subgroups. The indirect effects were evaluated using *n* = 5000 bootstrap samples. Further, to rule out effects of heteroscedasticity, the analyses used the HC3 estimator for heteroscedasticity-consistent standard errors. The significance of the indirect effects was tested by using 95% confidence intervals.

To test for the robustness of the mediation analyses and to rule out possible and previously shown influences of age, BMI, and the current relationship-status on mediators and mental health outcomes (e.g., Willitts et al., 2004; McLaren et al., 2008; Kessler et al., 2010), all mediation analyses were subsequently repeated, controlling for the above mentioned variables as covariates.

Overall, the significance level was defined as *p* < 0.05. To classify the effect size of possible gender differences, Hedge’s *g* for different group sizes was calculated. Conventions for *g* are that values of *g* ≥ 0.20 indicate small effects, values of *g* ≥ 0.50 indicate medium, and values of *g* ≥ 0.80 indicate large effects (Cohen, 1988).

## Results

### Sample Characteristics

The final analysis sample (*N* = 501; 407 female, 94 male) had an overall age range from 18 to 74 years with a mean age of 31.2 years (*SD* = 11.7) and a mean BMI in the normal weight range (*M* = 23.9 kg/m^2^, *SD* = 5.5 kg/m^2^). The majority of the sample was in a relationship with a partner (65.7%) and currently working (68.7%). Overall, 42.7% of the sample were students, 54.7% were employed, 7.8% were self-employed, 4.4% were jobless or incapable of working, 1.6% were retired (multiple answers were possible). The education level of the sample was generally high: 78.4% of the participants had a university entrance diploma, 12% had an advanced college entrance qualification, 8.2% had a secondary school certificate and 1.4% had other qualifications. Almost half of the sample (44.5%) reported that they had experienced severe teasing, bullying, or mobbing at least once in their lives.

### Gender Group Differences

A detailed overview on gender differences is provided in Tables 1, 2. The male subgroup was slightly older and had a higher BMI than the female subgroup (*p*s < 0.031). However, the distribution of relationship status, school education, employment, and the ratio of any teasing/bullying experiences did not differ between genders (*p*s > 0.547).

**Table 1:** Sample characteristics of the female and male subgroup in sociodemographic data.

Variable		Women	Men	Test statistics
*n*		407	94	
		***M* (*SD*)**	***M* (*SD*)**	

Age (years)		30.60 (11.42)	33.50 (12.72)	***t*(499) =** -**2.17, *p* = 0.031**
Body Mass Index (kg/m^2^)		23.53 (5.69)	25.31 (4.24)	***t*(179.29) =** -**3.41, *p* = 0.001**
		***n* (%)**	***n* (%)**	

Relationship status	In a relationship	270 (66.3)	59 (62.8)	χ^2^(1) = 0.43, *p* = 0.547
	No relationship	137 (33.7)	35 (37.2)	
School education	Secondary school level I	34 (8.4)	7 (7.4)	χ^2^(3) = 0.90, *p* = 0.822
	Advanced college entrance qualification	51 (12.5)	9 (9.5)	
	University-entrance diploma (“Abitur”)	316 (77.6)	77 (81.9)	
	Other	6 (1.5)	1 (1.1)	
Employment	Current employment	281 (69.0)	63 (67.0)	χ^2^(1) = 0.15, *p* = 0.712
	No current employment (incl. students without side jobs)	126 (31.0)	31 (33.0)	
Teasing experiences	Yes	182 (44.7)	41 (43.6)	χ^2^(1) = 0.04, *p* = 0.908
	No	225 (55.3)	53 (56.4)	


*Boldface entries indicate significant gender group differences, two-sided *p* < 0.05. In case of unequal variances in Levene’s test, degrees of freedom were adjusted*.

**Table 2:** Gender differences in teasing frequency, body image, and mental health (mean, standard deviations, and test statistics).

Variable	Women	Men	Levene’s test	Test statistics
*n*	407	94		
Appearance-teasing frequency (POTS)	2.09	1.86	*F* = 8.02	***t*(166.63) = 2.47, *p* = 0.015**^a^
	(0.95)	(0.76)	*p* = 0.005	
Appearance concerns (DCQ)	8.23	5.77	*F* = 10.85	***t*(163.66) = 5.27, *p* < 0.001**
	(4.75)	(3.89)	*p* = 0.001	
Appearance-based rejection sensitivity (ARS-D)	12.63	8.46	*F* = 19.29	***t*(194.03) = 6.22, *p* < 0.001**
	(7.72)	(5.34)	*p* < 0.001	
Depression (PHQ-9)	7.77	5.44	*F* = 5.18	***t*(160.07) = 4.08, *p* < 0.001**
	(5.73)	(4.81)	*p* = 0.023	
Anxiety (GAD-7)	7.33	5.20	*F* = 5.91	***t*(157.29) = 4.29, *p* < 0.001**
	(5.03)	(4.30)	*p* < 0.001	
Self-Esteem (RSES)	21.29	23.12	*F* = 4.11	***t*(156.71) = -2.78, *p* = 0.006**
	(6.49)	(5.58)	*p* = 0.043	


*Boldface entries indicate significant differences in group means, two-sided *p* < 0.05. In case of unequal variances in Levene’s test, degrees of freedom were adjusted. ^*a*^Group difference not significant in non-parametric test; POTS, perceptions of teasing scale; DCQ, dysmorphic concern questionnaire; ARS-D, appearance-based rejection sensitivity scale; PHQ-9, patient health questionnaire-9 - depression module; GAD-7, patient health questionnaire – anxiety module; RSES, rosenberg self-esteem scale*.

The *t*-test indicated that the frequency of early appearance-related teasing experiences (POTS-TF) in childhood and adolescence was higher in women compared to men. However, the effect size of the group difference was small (*g* = 0.25), and the group difference was no longer significant when applying non-parametric tests on group differences (*Z* = -1.84, *p* = 0.066). For the other appearance-related variables, dysmorphic concerns and ARS were higher in women compared to men with medium effect sizes (DCQ: *g* = 0.54; ARS-D: *g* = 0.57).

With regard to mental health variables, women showed significantly higher degrees of depression (*g* = 0.42), anxiety (*g* = 0.43), and lower self-esteem (*g* = 0.29) than men, with small to medium effect sizes. Non-parametric analyses confirmed the significant gender differences in these variables (all *p*s < 0.013).

### Correlations

As displayed in Table 3, the non-parametric correlation analyses in the female subgroup showed medium-sized relationships of appearance teasing frequency (POTS-TF) with dysmorphic concerns and with ARS (*p*s < 0.001). Further, the relationships of the POTS-TF and the three mental health variables were highly significant (*p*s < 0.001). Additionally, the appearance-related mediator variables and the mental health variables showed high interrelationships (*r*s > 0.52, *p*s < 0.001).

**Table 3:** Gender-specific correlations between teasing, body image, and mental health variables and gender differences in correlations.

Variable	1	2	3	4	5	6	Gender differences (POTS – Variable)
(1) Teasing frequency (POTS)	-	0.41**	0.32*	0.17	0.20	-0.20	
(2) Dysmorphic concerns (DCQ)	0.45**	-	0.37**	0.24	0.26	-0.24	*Z* = 0.42, *p* = 0.336
(3) Appearance-based rejection sensitivity (ARS-D)	0.38**	0.52**	-	0.06	0.07	-0.26	*Z* = 0.59, *p* = 0.278
(4) Depression (PHQ-9)	0.38**	0.51**	0.43**	-	0.72**	-0.48**	*Z* = 1.97, *p* = 0.025^∗^
(5) Anxiety (GAD-7)	0.37**	0.49**	0.40**	0.77**	-	-0.39**	*Z* = 1.60, *p* = 0.055
(6) Self-Esteem (RSES)	-0.30**	-0.44**	-0.50**	-0.61**	-0.60**	-	*Z* = -0.92, *p* = 0.179


*Correlation coefficients are based on Spearman’s correlation, two-sided; Values below the diagonal base on the female subgroup, values above the diagonal refer to the male subgroup. POTS, perceptions of teasing scale; DCQ, dysmorphic concern questionnaire; ARS-D, appearance-based rejection sensitivity scale; PHQ-9, patient health questionnaire-9 – depression module; GAD-7, patient health questionnaire – anxiety module; RSES, modified Rosenberg self-esteem scale. Indications of significance base on Bonferroni-corrected *p*-values; Gender differences regarding the correlations of POTS and other variables were assessed via Fisher’s-Z-test; ^∗^*p* < 0.05; ^∗∗^*p* < 0.01*.

In the male subsample, appearance teasing frequency also showed medium relationships with dysmorphic concerns and ARS (*p*s < 0.010). However, the correlations of POTS-TF with mental health variables were small (*r*s ≤ 0.20) and not significant after Bonferroni-corrections (*p*s > 0.250).

The direct comparisons of the gender-specific correlation coefficients via Fisher-*Z*-tests showed that the relationship of teasing and depression was significantly higher in women than in men (*p* = 0.025), and there was a trend towards significance for anxiety (*p* = 0.055). The size of the correlation coefficients for teasing frequency and body image variables or self-esteem did not differ significantly between males and females.

### Mediation Analyses

**FIGURE 2:**
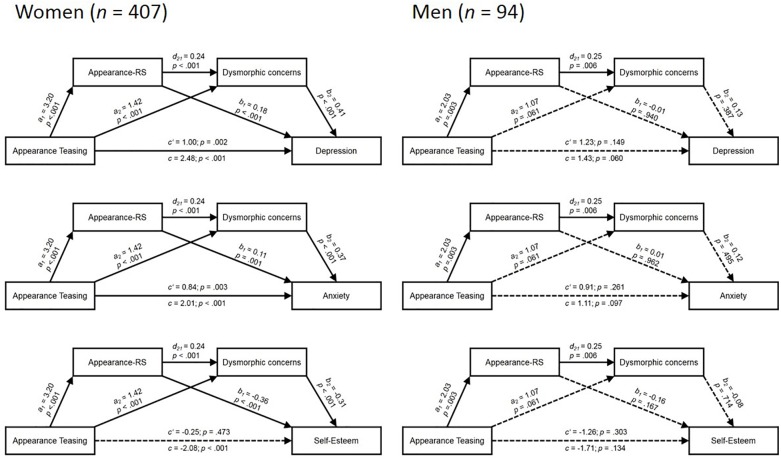
Gender-specific serial mediation models. Appearance-RS, appearace-based rejection sensitivity.

The mediation models for the female and male subsamples are displayed in Figure 2, and model indices are depicted in Table 4. For the female subsample, mediation analyses showed that early appearance teasing increased current ARS (*a_1_* = 3.20, *p* < 0.001) and that both, early teasing (*a_2_* = 1.42, *p* < 0.001) and ARS (*d_12_* = 0.24, *p* < 0.001) predicted dysmorphic concerns. In addition, dysmorphic concerns and ARS were significant mediators for the effects of early appearance teasing frequency on all three mental health variables.

**Table 4:** Comparison of gender-specific model indices for the total effect and serial mediation models in the female and male subgroups.

*Model characteristics*	Female (*n* = 407)				Male (*n* = 94)			
	*F*	*df*	*p*	*R*^2^	*F*	*df*	*p*	*R^2^*
A-Teasing → Depression	57.9	1, 405	<0.001	0.17	3.6	1, 92	0.060	0.05
A-Teasing → ARS → DC → Depression	67.1	3, 403	<0.001	0.37	1.5	3, 90	0.223	0.06
A-Teasing → Anxiety	48.0	1, 405	<0.001	0.14	2.8	1, 92	0.097	0.04
A-Teasing → ARS → DC → Anxiety	51.8	3, 403	<0.001	0.31	1.3	3, 90	0.283	0.05
A-Teasing → Self-Esteem	32.4	1, 405	<0.001	0.09	2.3	1, 92	0.133	0.05
A-Teasing → ARS → DC → Self-Esteem	58.2	3, 403	<0.001	0.35	1.7	3, 90	0.179	0.08


*A-Teasing, appearance teasing frequency (perceptions of teasing scale); ARS, appearance-based rejection sensitivity; DC, dysmorphic concerns*.

For the prediction of current degrees of depressive symptoms in the female subsample, early appearance teasing frequency was a significant and positive predictor (*c* = 2.48, *p* < 0.001) in the total effect model without consideration of the mediators. However, the explained variance increased by Δ*R^2^* = 0.20 when the mediators, dysmorphic concerns and ARS, were included in the model. All three possible indirect effects were significant [POTS-TF → ARS-D → PHQ-9: *b* = 0.58, 95% CI (0.31; 0.92); POTS-TF → ARS-D → DCQ → PHQ-9: *b* = 0.32, 95% CI (0.20; 0.50); POTS-TF → DCQ → PHQ-9: *b* = 0.58, 95% CI (0.37; 0.86)]. Correspondingly, the total indirect effect was significant [*b* = 1.48, 95% CI (1.09; 1.92)], whereas the direct effect was reduced by inclusion of the mediators but remained significant, too [*c’* = 1.00, *p* = 0.002; 95% CI (0.38; 1.62)].

For the degree of anxiety symptoms in women, the pattern was comparable: Early appearances teasing experiences significantly predicted higher current anxiety (*c* = 2.01, *p* < 0.001) in the total effect model. Inclusion of the appearance-related mediators increased the explained variance by Δ*R^2^* = 0.17. Again, all three indirect effects were significant [POTS-TF → ARS-D → GAD-7: *b* = 0.35, 95% CI (0.15; 0.61); POTS-TF → ARS-D → DCQ → GAD-7: *b* = 0.29, 95% CI (0.18; 0.44); POTS-TF → DCQ → GAD-7: *b* = 0.53, 95% CI (0.34; 0.79)]. The total indirect effect [*b* = 1.18, 95% CI (0.87; 1.54)] was stronger than the total direct effect [*c’* = 0.84, *p* = 0.003; 95% CI (0.29; 1.38)]. However, the direct effect remained significant.

With regard to self-esteem, we observed a slightly different pattern. While the total effect model showed that early appearance teasing experiences had a significant negative impact (*c* = -2.08, *p* < 0.001) on the female participants’ self-esteem, indirect effects of the mediators were even stronger for this mental health outcome. When including the mediators, the amount of explained variance increased by Δ*R^2^* = 0.26. Again, each of the three indirect effects was significant [POTS-TF → ARS-D → RSES: *b* = -1.14, 95% CI (-1.60; -0.77); POTS-TF → ARS-D → DCQ → RSES: *b* = -0.24, 95% CI (-0.40; -0.13); POTS-TF → DCQ → RSES: *b* = -0.44, 95% CI (-0.72; -0.24)]. In line with this, the total indirect effect was significant, *b* = -1.83; 95% CI [-2.39; -1.36]. However, as a result of the included mediators, the direct effect of appearance teasing on self-esteem was no longer significant [c’ = -0.25, *p* = 0.473; 95% CI (-0.93; 0.43)].

For the male subsample, the results differed from those of the female group. Early appearance teasing increased ARS (*a_1_* = 2.01, *p* = 0.003). Besides, ARS (*d_12_* = 0.25, *p* = 0.006), but not appearance teasing (*a_2_* = 1.07, *p* = 0.061), predicted dysmorphic concerns. However, there were neither significant direct effects of early appearance teasing experiences on current mental health outcomes, nor any significant indirect effects via the mediators. ARS-D and DCQ values predicted none of the mental health outcomes in the serial mediation models (*p*s > 0.167).

The total effect models were not significant for depression, anxiety, and self-esteem in the male subgroup. Here, the amount of increased explained variance never surpassed Δ*R^2^* = 0.03 when the appearance-related mediators were included in the regression model (see Table 4). Further, none of the indirect effects via any of the mediators was significant [total indirect effects: *Y* = PHQ-9: *b* = 0.19, 95% CI (-0.29; 0.88); *Y* = GAD-7: *b* = 0.19, 95% CI (-0.30; 0.88); *Y* = RSES: *b* = -0.45, 95% CI (-1.12; 0.20)].

### Control Analyses

We re-conducted all serial mediation analyses while controlling for possible influences of BMI, age, and relationship-status (binary code: 0 = no relationship; 1 = relationship) as covariates. The pattern of results remained stable in those analyses. There were no changes in the significance levels or effects of any of the aforementioned models, predictors, or mediators.

## Discussion

In the present study, we examined gender differences in appearance teasing, its relationship with two body image variables (ARS and dysmorphic concerns), and associations with different mental health outcomes – depression, anxiety, and self-esteem. Against the background of previous etiological models for body image disorders (e.g., Neziroglu et al., 2008) and recent empirical findings (e.g., Lavell et al., 2014; Webb et al., 2015; Densham et al., 2017), we tested potential serial mediation effects of ARS and dysmorphic concerns in the relationship of appearance teasing and mental health. A focus of these investigations was an analysis of potential gender-specific differences.

Overall, the ratio of men and women reporting any teasing events did not differ significantly. This finding contradicts previous reports on higher prevalence of appearance teasing in girls and women compared to boys and men (Farrow and Fox, 2011; Almenara and Ježek, 2015). However, in line with results from previous research, women showed higher impairments in body image variables (e.g., Feingold and Mazzella, 1998; Demarest and Allen, 2000; Buhlmann et al., 2010), as well as stronger mental health impairments and lower self-esteem compared to men (Piccinelli and Wilkinson, 2000; McMullin and Cairney, 2004; Bleidorn et al., 2016; von Soest et al., 2016).

Regarding the bivariate relationships of early teasing experiences with body image variables, we observed that appearance teasing frequency was significantly associated with higher degrees of adult ARS and stronger dysmorphic concerns in both, men and women. These findings support assumptions about the long lasting impact of early victimization experiences, especially with regard to body image (Eisenberg et al., 2003; Menzel et al., 2010; Liang et al., 2011; Webb et al., 2015). For the relationship between appearance teasing and mental health, effects were less pronounced in men compared to women. Thus, men seem to experience fewer mental health impairments because of early appearance teasing, confirming earlier findings (e.g., Gruber and Fineran, 2008; Carbone-Lopez et al., 2010; Feragen and Stock, 2016). In addition, the associations of body image variables and mental health were strong in women, but weak and not significant in men. Again, these findings are in line with previous research (Furnham et al., 2002; McMullin and Cairney, 2004).

In the following exploration of the serial mediation model, we observed additional effects that contribute to the knowledge about potential pathways in the relationship of appearance teasing on mental health: One observation concerns a gender-invariant mediation effect in the first part of the model (POTS → ARS → DCQ). For both, men and women, ARS played a crucial role in predicting dysmorphic concerns elicited by appearance teasing. These results confirm previous findings regarding the important role of the dispositional trait of ARS as a mediator in the development of dysmorphic concerns and BDD symptoms (Park et al., 2010; Lavell et al., 2014; Webb et al., 2015; Densham et al., 2017). Thus, the findings highlight the important role of ARS in the initiation and perpetuation of body image disorders. The construct could therefore be a factor to explore for potential inclusion in advanced etiological models of body image disorders - independent of gender.

However, the present study suggests that there are in fact gender differences regarding the other pathways of the serial mediation model: In women, appearance teasing experiences were significantly related to current depression, anxiety, and low self-esteem. This relationship is further explained by mediation effects of ARS and dysmorphic concerns. Thus, early appearance teasing increases ARS with its enhanced fear of interpersonal rejection due to the appearance. ARS then fosters enhanced dysmorphic concerns (i.e., excessive worries about the own appearance). These worries then increase the likelihood of depressive and anxious feelings as well as lower self-esteem in adulthood. Especially for self-esteem in adult women, early appearance teasing no longer served as a predictor when the two body image mediators were included in the analysis. This serial mediation effect explains how ARS and dysmorphic concerns are phenomena that contribute to mental health issues in women. It further shows that appearance and body image still strongly contribute to self-esteem in women (Furnham et al., 2002; Calogero et al., 2007; Swami et al., 2010). This effect may be caused by the stronger internalization of beauty-ideals for female individuals (e.g., Vandenbosch and Eggermont, 2012) and stronger external exposure to exaggerated beauty standards (Grabe et al., 2008). Also, women have been shown to more readily remember appearance teasing than competency teasing (Agliata et al., 2007). This cognitive accessibility could constitute another vulnerability factor for the continued negative effects of early appearance teasing experiences on the mental health of adult females.

In the male subsample, the overall pattern was different: Despite of negative effects on ARS and dysmorphic concerns, early appearance teasing was not significantly related to any of the mental health variables. In addition, the body image variables were not associated with mental health outcomes in men. This shows, that although appearance teasing has specific negative effects on body image, these effects do not generalize to general mental health outcomes in men, constituting a substantial gender difference.

Several explanations can be consulted for this gender difference: male mental health might generally be comprised of more sources of self-esteem, such as academic, financial, or athletic performances (Bolognini et al., 1996), limiting the impact of body image and of appearance teasing on mental health. Thus, it would be interesting to examine, whether teasing in other domains (e.g., competency) has stronger effects on adult mental health in men.

Another explanation may lie in predominant tendencies for externalizing behaviors rather than internalizing concerns in response to victimization (Llewellyn and Rudolph, 2014; Gregg et al., 2016; Stubbs-Richardson et al., 2018). Boys may be more prone to cope with victimization by showing dysfunctional behaviors (e.g., substance use, aggressive behaviors), or perpetuate victimization to third persons. In contrast, girls show more avoidance behaviors (Llewellyn and Rudolph, 2014), practice self-blame, and self-harming eating behaviors (Haines et al., 2006; Ata et al., 2007).

In addition, gender differences in coping abilities could contribute to the observed effects (Tamres et al., 2002; Boulton, 2013). For example, women tend to use more rumination, a dysfunctional emotion regulation strategy that can explain gender differences in mental health (e.g., Aldao et al., 2010; Opwis et al., 2017). Women also tend to seek social support as a strategy to cope with emotional and social stressors (Tamres et al., 2002). Therefore, losing this resource due to elevated ARS specifically infringes available coping strategies that might help precluding mental health problems in women. The identification of these possible resilience and vulnerability factors that moderate gender-specific appearance teasing effects should be subjects of future studies.

With regard to therapeutic implications, the present findings highlight the need to address ARS in body image treatments in both genders, for example via behavioral experiments and cognitive restructuring. Especially for women, therapeutic work with ARS could indirectly improve dysmorphic concerns and associated general mental health. Behavioral experiments may be useful therapeutic tools to generate disconfirming evidence regarding fear of interpersonal rejection. In addition, the results hint at the importance of more prevention measures to avoid long term negative outcomes of appearance teasing in women. Interventions that establish more sources of self-esteem beyond appearance at an early age might enhance resilience in women. In addition, cognitive reframing of teasing experiences could help preventing mental health impairments in female victims of appearance teasing.

Despite of these promising results, the study is subject to several limitations. For example, the data in this study is cross-sectional and bases on a convenience sample with a larger proportion of female compared to male respondents. The known restrictions of online surveys apply to this study (e.g., a lack of control over the circumstances while filling in the questionnaire and possible biases regarding the sample; see Barratt et al., 2015). We controlled for possible confounding factors, such as extreme response latencies, with conservative data screening procedures. Still, we cannot rule out any probabilities of sampling biases, especially with regard to the few inclusion and exclusion criteria applied. For example, because of the announcement of the study as research on appearance, body image, and mental health, it is possible that there was a selection bias. The sample may be characterized by a special interest in these topics. Future studies should therefore particularly ensure the recruitment of representative samples. Further, we were not able to control for possible confounding effects of prior or current interventions (e.g., medication or psychotherapy) on the mental health status of the respondents.

Limitations also result from the retrospective reports of teasing. We cannot rule out the possibility of distorted reports of teasing due to the long latencies and possible memory biases, especially in older participants. Despite of our control analysis for age, this limitation has to be taken into account when appraising the findings of this study. Future studies on this topic should also aim at recruiting equal sample sizes for the male and female subgroups to ensure equal statistical power to detect significant effects in men and women. In general, this cross-sectional study constitutes a first step in understanding the gender-specific relationship of appearance teasing, body image variables, and mental health. Actual experimental and longitudinal research (e.g., with applications of ecological momentary assessment) should to be aspired to allow for veritable analyses of mechanistic processes in this research field.

Further, we did not assess sexual orientation. Therefore, we cannot provide answers about possible differences regarding the effects of appearance teasing on body image and mental health outcomes with respect to hetero-, homo-, bi-, or transsexual individuals. Earlier research has shown more victimizations and an increased importance of body image in sexual minorities, such as homosexual men (e.g., Gruber and Fineran, 2008; Frederick and Essayli, 2016). Therefore, future studies should take into account the possible moderating effects of sexual orientation in explanatory models.

However, the present study also has several strengths: It directly compares gender differences of appearance teasing and its effects on adult mental health. Earlier studies often focused on effects in adolescence and/or examined effects in female samples only. In addition, to our best knowledge, this was the first study to examine more complex mediation pathways regarding the role of two relevant body image variables, ARS as a dispositional factor and dysmorphic concerns as a cognitive factor, in explaining gender-specific effects of appearance teasing on various mental health outcomes. In addition, we tested all mediation models again, while controlling for potential influences of age, BMI, and relationship-status and obtained the same pattern of results. Thus, the effects have proven stable against possible influences of those common confounders with respect to body image, as well as mental health variables.

## Conclusion

The present study observed gender-specific effects of early appearance teasing experiences on mental health in adulthood, although men and women reported similar frequencies of teasing. While appearance teasing experiences have specific negative effects on the body image of both, women and men, the negative effects do not stretch out to more global mental health concerns in men. However, in women, ARS and dysmorphic concerns play an important mediating role in explaining negative effects of appearance teasing in childhood and adolescence on mental health in adulthood. The study highlights the role of ARS as an important construct in the etiology of body image disorders that should therefore be a specific target of therapeutic interventions for this array of mental disorders. However, future research needs to explore moderators that serve as resilience and vulnerability factors regarding gender-specific negative effects of appearance teasing on mental health.

## Data Availability

The raw data supporting the conclusions of this manuscript will be made available by the authors, without undue reservation, to any qualified researcher.

## Ethics Statement

This study was carried out in accordance with the recommendations of “Richtlinien der Ethik-Kommission der Bergischen Universität Wuppertal.” All subjects gave written informed consent in accordance with the Declaration of Helsinki. The protocol was approved by the Ethik-Kommission der Bergischen Universität Wuppertal (Reference No. MS/JE 180312 Schmidt 2).

## Author Contributions

JS drafted the manuscript with important contributions from AM, designed the study in collaboration with AM, and conducted the analyses. Both authors have approved the final manuscript to be published.

## Conflict of Interest Statement

The authors declare that the research was conducted in the absence of any commercial or financial relationships that could be construed as a potential conflict of interest.
